# The Increasing Vulnerability of South Asians in Canada during the COVID-19 Pandemic

**DOI:** 10.3390/ijerph19052786

**Published:** 2022-02-27

**Authors:** Tijhiana Rose Thobani, Zahid Ahmad Butt

**Affiliations:** School of Public Health Sciences, University of Waterloo, Waterloo, ON N2L 3G1, Canada; trthoban@uwaterloo.ca

**Keywords:** South Asian, COVID-19, Canada, disproportionate

## Abstract

Canadian South Asians are being economically, socially, politically, and culturally impacted by the COVID-19 pandemic. There is currently a gap in the literature on the unique challenges faced by this specific group of individuals. People of color and ethnic minorities are being homogenized in the media and throughout the literature when addressing populations disproportionally impacted by the current situation. This commentary aims to add a new perspective to the current literature by specifically exploring factors that may contribute to the high rates of COVID-19 among South Asian communities in Canada. Another goal is to highlight the importance of providing tailored support and attention for this community and the negative consequences if this is not correctly done. Factors such as overrepresentation in essential work and financial instability are discussed. Pre-existing health conditions among South Asians such as diabetes, hypertension, anxiety, and mood disorder are considered, as well as how the history of these conditions within this population elevates the risk of severe health complications. This commentary presents suggestions for addressing this gap in research, as well as directions for future public health initiatives and policies.

## 1. Introduction

South Asians in Canada are disproportionately experiencing the negative effects of the COVID-19 pandemic, compared to the general Canadian population [1]. According to Census Canada, South Asians represent the largest visible minority in the country [2]. Of the Canadian population, this group is reported to account for almost 5%, and this percentage is increasing [3]. In this context, South Asians are individuals with an ethnic ancestry from India, Pakistan, Sri Lanka, Bangladesh, or Nepal [2]. The media have placed a spotlight on this community as being non-compliant with public health guidelines and has blamed South Asians for being culprits in transmitting the virus because of ‘cultural practices’, such as large cultural festivities [4]. However, limited research has been done to explore the reality of high virus transmission by this community. Financial instability and employment in essential work are driving factors for COVID-19 spread; many South Asians in Canada are engaged in the frontlines of essential work, yet this has failed to be discussed in the literature [1,5]. Some of the highest rates of COVID-19 transmission have occurred among racial groups in Canada, compared to the rest of the population [6]. South Asians, in particular, have been severely hit by the negative effects of the pandemic, in terms of both the virus and socio-economic consequences [6]. It has been determined that the death rate in this community is around 25% greater in neighborhoods highly populated with South Asians, compared to communities with a smaller South Asian population [1]. In Toronto alone, 80% of COVID-19 infections were linked to visible racial minorities; data showed that South Asians were disproportionately represented in this statistic in relation to other racial minority groups [7]. The media have created a distorted image that this population has these high rates of COVID-19 because of failure to follow the established protocol; this is far from reality [4]. However, their high rates of unemployment and their overrepresentation in essential work may lead to a higher risk of contracting the virus. The aim of this commentary is to explore factors that may contribute to the high rates of COVID-19 among South Asian communities in Canada and understand how the pandemic has exacerbated the existing socio-economic disparities of this vulnerable community. The commentary will conclude by highlighting areas of opportunity for future research.

## 2. Unemployment and Overrepresentation in Essential Work

After Indigenous communities, South Asians in Canada have the highest rate of unemployment; over 17% of this population is documented as unemployed [3]. The Association for Canadian Studies conducted a national survey that revealed that three-quarters of South Asians are personally threatened by the current economic crisis, making them Canada’s most vulnerable racialized group [8]. Although financial support—the Canadian Emergency Response Benefit—was made available to the population by the Federal government, many South Asians did not qualify because of their immigration status [8]. As a result, many individuals from this population are overrepresented in low-wage essential work [8]. This includes places where measures established by the public health protocol such as social distancing cannot always be implemented, such as grocery stores, public transportation, health care, and factories. In Brampton, Ontario, employees of workplaces such as Amazon and food processing warehouses are disproportionately staffed by immigrants, with many of them being from South Asia [9]. It took an outbreak of 240 cases of COVID-19 in a single week to eventually shut down for two weeks the Amazon warehouse [9]. The Brampton trucking sector comprises approximately 181,000 drivers who are identified as South Asian [4]. These drivers are continuously driving in and out of hotspot areas, again increasing their exposure to the virus [4]. Comparing this to other truckers in Canada on a larger scale, one in five Canadian truck drivers are South Asians [10]. When looking at Vancouver and Toronto specifically, almost 50% of truck drivers are South Asian [10]. A prospective cohort study examining COVID-19 spread and essential work among the BIPOC (Black, Indigenous, and other peoples of color) communities found that they are at a greater risk of contracting the virus because of their working conditions [11]. Due to differences in power dynamics, their employers’ fears have left them continue working in unsafe conditions [4].

## 3. Vaccine Uptake

Compared to the Canadian white population, Canadian South Asians have a five to ten-fold greater risk of contracting the COVID-19 virus [12]. In fact, 82.5% of this population is willing to the receive the vaccine; however, many people from this group have yet to receive the vaccine [13]. Individuals from this community experience hesitancy to accept the COVID-19 vaccination; this is concerning, as many of them experience socio-cultural factors that increase their risk of contracting the disease [12]. A prospective cohort study titled “COVID CommUNITY–South Asian” is currently recruiting individuals in Canada who identify as South Asian [12]. This study will allow understanding how socioeconomic, cultural, and other non-biomedical factors contribute to vaccine access and hesitancy in the community [12].

## 4. Pre-Existing Health Concerns and Comorbidities within This Community

The Canadian South Asian population is at a higher risk of developing comorbidities and of falling seriously ill from COVID-19 [14,15]. For example, South Asians living in Canada have higher incidence and prevalence of cardiovascular disease and twice the prevalence of diabetes than the white population [15]. This group presents a genetically based greater resistance to insulin and increased levels of cholesterol. Diabetes, hypertension, and heart disease, which are all highly present among this community, act as co-morbid conditions with COVID-19 [14]. High rates of morbidity and mortality as a result of COVID-19 will continue to affect this community if action is not taken to support them.

Connections have been made between the pandemic and negative mental health outcomes [16]. A study that examined mental health concerns among the Canadian South Asian population reported increased levels of anxiety disorders, severe stress, and mood disorders [2]. Factors such as socioeconomic status, food security status, and immigration timing were found to contribute to mental health concerns present among these individuals [2]. Being aware of the pre-existing mental health disorders in this community is crucial, especially with the elevated pressures of the pandemic. Essential workers in the pandemic have reported greater occurrences of poor mental health and decline in their mental well-being [16]. South Asians account for a significant portion of essential workers in Canada, suggesting that this community needs that direct and tailored attention be given to their mental health and well-being during the COVID-19 pandemic.

## 5. Future Directions

Accurate and robust data must be collected to assess the representation of this ethnic minority in essential services as well as to understand the underlying factors that contribute to the increased risk of COVID-19 among South Asians. In addition, support for these individuals should be provided in a way that is beneficial to and supportive of cultural practices [6]. There is currently a gap in research on the effects of the COVID-19 pandemic among Canadian South Asians. There is limited knowledge on why this community experiences higher rates of COVID-19 and severe symptoms of the virus compared to the general population. Steps must be taken to immediately address this gap and control the rapid spread of the virus among this community [1]. This does not simply mean communicating public health guidance in a different language, but rather understanding how cultural and contextual factors affect the ability of this community to practice social distancing [1,17]. It is also necessary to perform a more robust data collection, distinguishing between ethnic groups being diagnosed with COVID-19 and determining how many individuals experience hospitalization and death [17]. This is important to ensure that strategies to contain the virus are tailored to the needs of specific ethnic groups. The connection between essential work and South Asians needs to be better defined and addressed.

The Canadian Government should consider extending financial support similar to the Canadian Emergency Response Benefit or including immigrant workers in the current criteria [18]. Workers from this marginalized community face an increased risk of contracting the COVID-19 virus and are discriminated against by being excluded from Canadian financial support [18]. Moving forward, there should be financial support for this group, regardless of the immigration status, to thus minimize the need for them in essential work [18]. Addressing this inequality through legalizing these immigrants would provide them with the opportunities offered to other Canadians and with access to safe job options.

When making future decisions and interventions to address the high rates of COVID-19 among the South Asian community, sociocultural factors must be taken into consideration [19]. For example, individuals from this community have cultural and religious reasons from not being vaccinated, which makes cultural competency important when moving toward addressing this concern [19]. Taking actions such as working with community and faith leaders to communicate the importance of vaccination can be done to address the spread of COVID-19 in the community [19].

There is also minimal knowledge on how the results of the pandemic have impacted the mental health and well-being in this community. At this point in time, there are conflicting articles in the media on the spread of COVID-19 within this group [4]. This lack of knowledge must be addressed to better understand the root causes of their vulnerability and susceptibility to the effects of the coronavirus pandemic in Canada. This information can then be used to develop more inclusive public health campaigns addressing the unique conditions of this group. This can also be useful to develop meaningful future policies that consider the specific challenges of this group financially, socially, and mentally, as well as other social determinants of health [20,21].

## 6. Conclusions

In summary, attention needs to be given to the unique challenges faced by Canadian South Asians during the pandemic. This group of individuals are facing difficulty in accessing financial government support, work in unsafe conditions, and are largely represented in the country’s unemployment records. Given the high occurrence of pre-existing comorbidities and mental health conditions, specific assistance should be provided to this community. There is a pressing need for more data on the unique experience of this group, to better understand how and why COVID-19 is highly prevalent within this population. Public health messaging, policy development, and workplace environments must all consider the cultural, political, economic, and social factors that shape the experience of Canadian South Asians and how these factors influence their health outcomes in relation to COVID-19.

## Data Availability

No new data were created or analyzed in this study. Data sharing is not applicable to this article.
